# Crystal structures of the 1:1 salts of 2-amino-4-nitro­benzoate with each of (2-hy­droxy­eth­yl)di­methyl­aza­nium, *tert*-but­yl(2-hy­droxy­eth­yl)aza­nium and 1,3-dihy­droxy-2-(hy­droxy­meth­yl)propan-2-aminium

**DOI:** 10.1107/S2056989018015578

**Published:** 2018-11-09

**Authors:** James L. Wardell, Edward R. T. Tiekink

**Affiliations:** a Department of Chemistry, University of Aberdeen, Old Aberdeen, AB24 3UE, Scotland; b Research Centre for Crystalline Materials, School of Science and Technology, Sunway University, 47500 Bandar Sunway, Selangor Darul Ehsan, Malaysia

**Keywords:** crystal structure, carboxyl­ate, mol­ecular salt, hydrogen-bonding

## Abstract

The crystal and mol­ecular structures of the title mol­ecular salts, C_4_H_12_NO^+^·C_7_H_5_N_2_O_4_^−^, (I), C_6_H_16_NO^+^·C_7_H_5_N_2_O_4_^−^, (II), and C_4_H_12_NO_3_^+^·C_7_H_5_N_2_O_4_^−^, (III), are described. The common feature of these salts is the presence of the 2-amino-4-nitro­benzoate anion, which exhibit non-chemically significant variations in the conformational relationships between the carboxyl­ate and nitro groups, and between these and the benzene rings they are connected to. The number of ammonium-N—H H atoms in the cations increases from one to three in (I) to (III), respectively, and this variation significantly influences the supra­molecular aggregation patterns in the respective crystals. Thus, a linear supra­molecular chain along [100] sustained by charge-assisted tertiary-ammonium-N—H⋯O(carboxyl­ate), hy­droxy-O—H⋯O(carboxyl­ate) and amino-N—H⋯O(carboxyl­ate) hydrogen-bonds is apparent in the crystal of (I). Chains are connected into a three-dimensional architecture by methyl-C—H⋯O(hy­droxy) and π–π inter­actions, the latter between benzene rings [inter-centroid separation = 3.5796 (10) Å]. In the crystal of (II), a supra­molecular tube propagating along [901] arises as a result of charge-assisted secondary-ammonium-N—H⋯O(carboxyl­ate) and hy­droxy-O—H⋯O(carboxyl­ate) hydrogen-bonding. These are connected by methyl­ene- and methyl-C—H⋯O(nitro) and π–π stacking between benzene rings [inter-centroid separation = 3.5226 (10) Å]. Finally, double-layers parallel to (100) sustained by charge-assisted ammonium-N—H⋯O(carboxyl­ate), ammonium-N—H⋯O(hy­droxy) and hy­droxy-O—H⋯O(carboxyl­ate) hydrogen-bonds are apparent in the crystal of (III). These are connected in a three-dimensional architecture by amine-N—H⋯O(nitro) hydrogen-bonds.

## Abstract 2

Three ammonium salts of 2-amino-4-nitro­benzoate are described. Based on N—H⋯O and O—H⋯O hydrogen-bonding involving the different constituents, supra­molecular chains, tubes and double-layers are found in their crystals.

## Chemical context   

Despite being tetra­morphic (Wardell & Tiekink, 2011 ▸; Wardell & Wardell, 2016 ▸), readily forming co-crystals (Wardell & Tiekink, 2011 ▸) and providing systematic series of crystals of alkali metal, *e.g*. Na^+^, K^+^ (Smith, 2013 ▸), Rb^+^ (Smith, 2014*a* ▸) and Cs^+^ (Smith & Wermuth, 2011 ▸), and ammonium salts, see below, studies of the relatively small benzoic acid derivative, 2-amino-4-nitro­benzoic acid, are still comparatively limited. Most crystallographic investigations of the acid have focused upon an evaluation of the hydrogen-bonding propensities occurring in derived ammonium salts of the 2-amino-4-nitro­benzoate anion. Thus, studies have been described with a range of salts, starting with the simplest, *i.e*. N^(+)^H_4_ (Smith, 2014*b* ▸), H_2_NN^(+)^H_3_ (Wardell *et al.*, 2017 ▸) and (H_2_N)_2_C=N^(+)^H_2_ (Smith *et al.*, 2007 ▸) to *R*_2_N^(+)^H_2_, *i.e. R* = Me, *n*-Bu (Wardell *et al.*, 2016 ▸), cyclohexyl (Smith *et al.*, 2004 ▸) and *R*_2_ = (CH_2_CH_2_)_2_O (Smith & Lynch, 2016 ▸), and more complicated ammonium cations such as 4-(4-acetyl­phen­yl)piperazin-1-ium (Jotani *et al.*, 2018 ▸) and the dication, H_3_N^(+)^CH_2_CH_2_N^(+)^H_3_ (Smith *et al.*, 2002 ▸). As a continuation of on-going inter­est in this area, the results of co-crystallization experiments between 2-amino-4-nitro­benzoic acid (*L*H) and amines substituted with hy­droxy groups, *i.e*. each of Me_2_N(CH_2_CH_2_OH), (*t*-Bu)N(H)CH_2_CH_2_OH and (HOCH_2_)_3_CNH_2_ are described whereupon the anhydrous 1:1 salts, *i.e*. [Me_2_N^(+)^H(CH_2_CH_2_OH)]*L* (I), [(*t*-Bu)N^(+)^H_2_(CH_2_CH_2_OH)]*L* (II) and [(HOCH_2_)_3_CN^(+)^H_3_]*L* (III), were isolated. Herein, a description of the crystal and mol­ecular structures of (I)–(III) are presented.
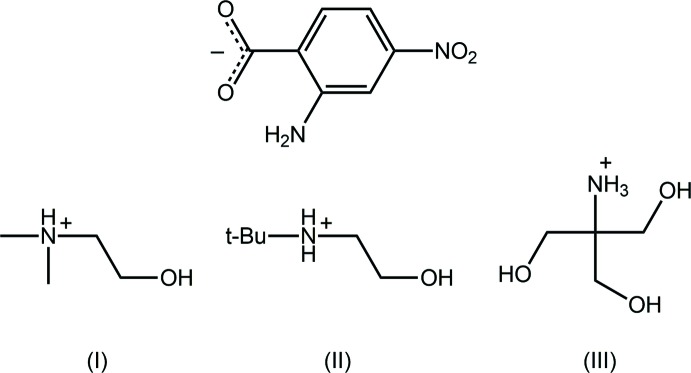


## Structural commentary   

**Figure 1:**
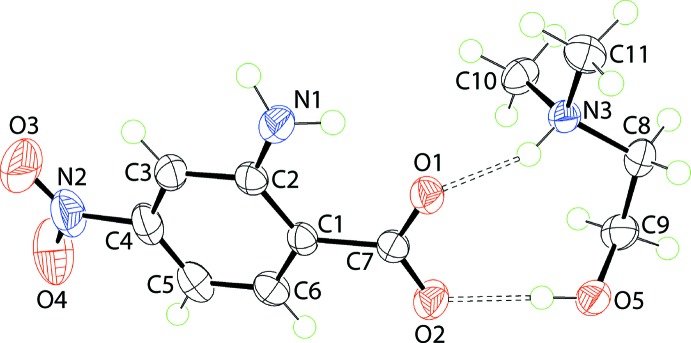
The mol­ecular structures of the ions comprising the asymmetric unit of (I) showing the atom-labelling scheme and displacement ellipsoids at the 70% probability level. Dashed lines indicate a hydrogen bonds.

The mol­ecular structures of the constituent ions in (I) are shown in Fig. 1 ▸ and selected geometric data for this and for (II) and (III), are collected in Table 1 ▸. That proton transfer occurred during co-crystallization is confirmed by the experimental equivalence of the C7 O1, O2 bond lengths of 1.270 (2) and 1.258 (2) Å, respectively, in the 2-amino-4-nitro­benzoate anion and in the pattern of hydrogen-bonding inter­actions, as described below in *Supra­molecular features*. In the anion, the carboxyl­ate group is tilted out of the plane of the benzene ring to which it is connected with the dihedral angle being 6.7 (3)°. Similarly, the nitro group lies out of the plane of the benzene ring, forming a dihedral angle of 6.6 (3)°. A dis-rotatory relationship between the carboxyl­ate and nitro substituents is indicated by the dihedral angle between them of 11.5 (4)°. An intra­molecular amine-N1—H⋯O1(carboxyl­ate) hydrogen-bond is noted which closes an *S*(6) loop, Table 2 ▸. In the Me_2_N^(+)^(H)CH_2_CH_2_OH cation, the N3—C8—C9—O5 torsion angle of −71.15 (19)° is indicative of a −syn-clinal conformation.

**Figure 2:**
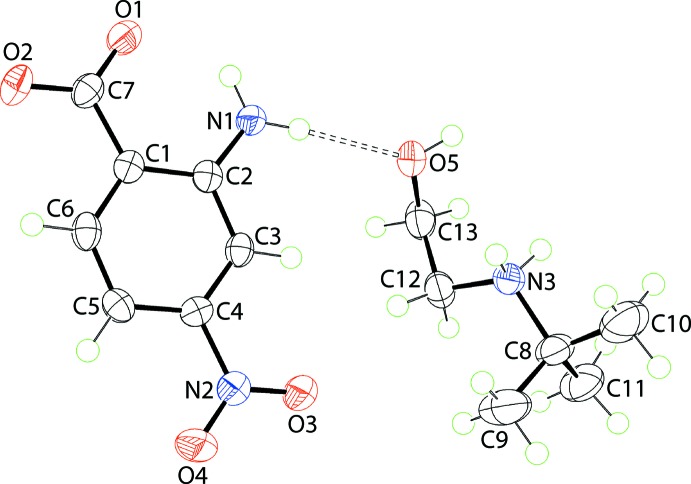
The mol­ecular structures of the ions comprising the asymmetric unit of (II) showing the atom-labelling scheme and displacement ellipsoids at the 70% probability level. The dashed line indicates a hydrogen bond.

The anion in (II), Fig. 2 ▸, presents essentially the same features as just described for (I), Tables 1 ▸ and 3 ▸, with the exception of the con-rotatory relationship between the carboxyl­ate and nitro substituents. The (*t*-Bu)N^(+)^H_2_(CH_2_CH_2_OH) cation is relatively rare, being reported for the first time in its salt with sulfa­thia­zolate only in 2012 (Arman *et al.*, 2012 ▸). As for the cation in (I), the N3—C12—C13—O5 torsion angle for the cation in (II) of −55.18 (18)° is indicative of a −syn-clinal conformation.

**Figure 3:**
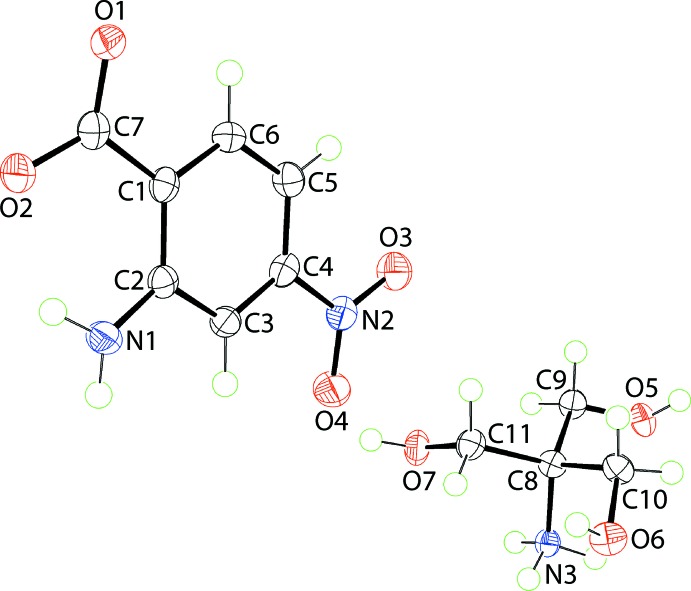
The mol­ecular structures of the ions comprising the asymmetric unit of (III) showing the atom-labelling scheme and displacement ellipsoids at the 70% probability level.

The anion in (III), Fig. 3 ▸, exhibits the greatest twist between the carboxyl­ate and benzene groups among the series but, a con-rotatory relationship between the carboxyl­ate and nitro substituents means the dihedral angle between them is not as great as in the anion of (I), Tables 1 ▸ and 4 ▸. The (HOCH_2_)_3_CN^(+)^H_3_ cation exhibits N3—C8—C9—O5, N3—C8—C10—O6 and N3—C8—C11—O7 torsion angles of −59.01 (18), −49.84 (19) and −58.12 (18)°, respectively, indicating −syn-clinal relationships.

## Supra­molecular features   

As expected from the chemical compositions of (I)–(III), significant charge-assisted hydrogen-bonding is apparent in their respective crystals. Geometric data characterizing these and other identified inter­actions are collated in Tables 2 ▸–4 ▸ ▸, respectively.

**Figure 4:**
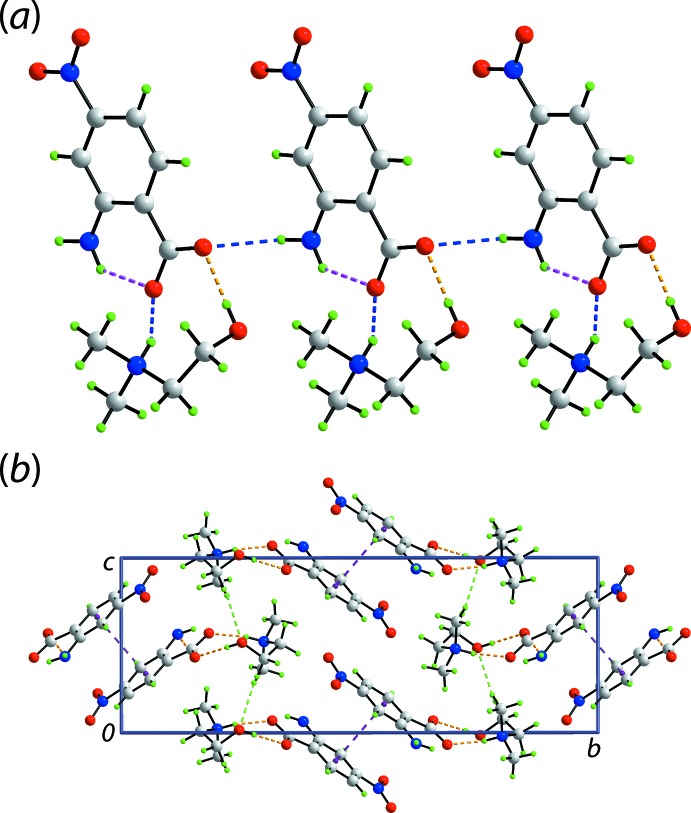
The mol­ecular packing in (I): (*a*) linear, supra­molecular chain along the *a* axis sustained by charge-assisted amine-N—H⋯O(carboxyl­ate) and hy­droxy-O—H⋯O(carboxyl­ate) hydrogen-bonding inter­actions shown as blue and orange dashed lines, respectively; intra­molecular amine-N—H⋯O(carboxyl­ate) hydrogen bonds are represented by pink dashed lines, and (*b*) a view of the unit-cell contents in projection down the *a* axis. The methyl-C—H⋯O(hy­droxy) and π–π inter­actions are shown as green and purple dashed lines, respectively.

As indicated in Fig. 1 ▸, the anion and cation in (I) are linked *via* charge-assisted ammonium-N3—H⋯O(carboxyl­ate) and hy­droxy-O—H⋯O(carboxyl­ate) hydrogen-bonds to form a nine-membered {⋯OCO⋯HNC_2_OH} heterosynthon. These are connected into a linear, supra­molecular chain along the *a*-axis direction *via* amino-N—H⋯O(carboxyl­ate) hydrogen-bonds, Fig. 4 ▸(*a*). The chains are linked along the *b* axis *via* π–π inter­actions between benzene rings [inter-centroid separation = 3.5796 (10) Å for symmetry operation: −*x*, −*y*, 1 − *z*], and methyl-C—H⋯O(hy­droxy) inter­actions link mol­ecules along the *c*-axis to consolidate the three-dimensional packing, Fig. 4 ▸(*b*).

**Figure 5:**
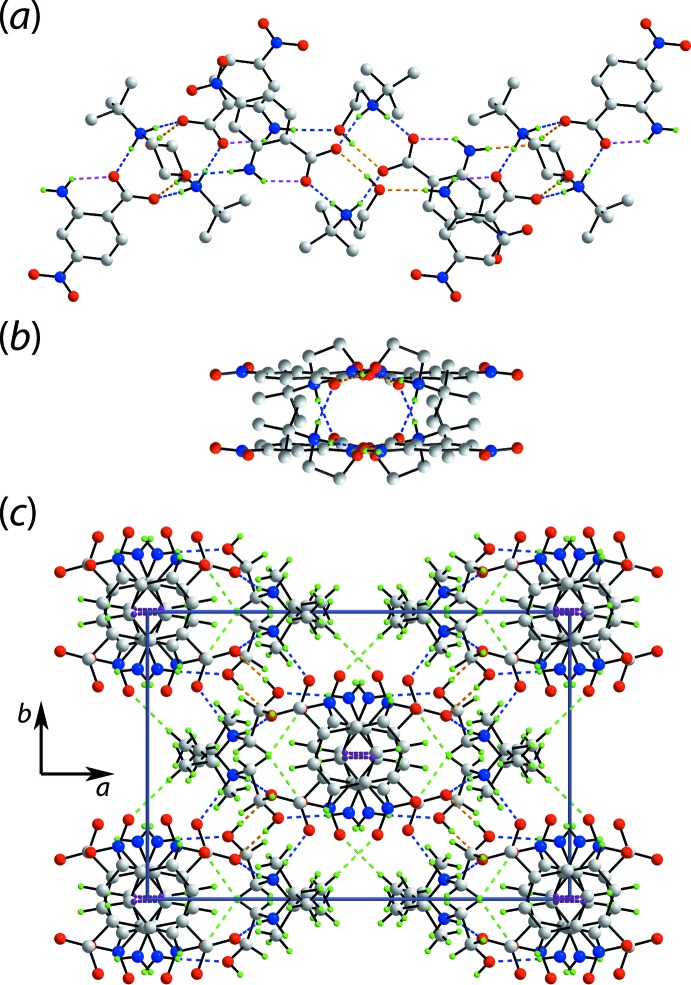
The mol­ecular packing in (II): (*a*) linear, supra­molecular tube along [901] sustained by charge-assisted amine-N—H⋯O(carboxyl­ate) and hy­droxy-O—H⋯O(carboxyl­ate) hydrogen-bonding inter­actions shown as blue and orange dashed lines, respectively; intra­molecular amine-N—H⋯O(carboxyl­ate) hydrogen bonds are represented by pink dashed lines, (*b*) end-on view of the supra­molecular tube and (*c*) a view of the unit-cell contents in projection down the *c* axis. The methyl­ene-, methyl-C—H⋯O(nitro) and π–π inter­actions are shown as green and purple dashed lines, respectively. In each of (*a*) and (*b*), non-participating H atoms are omitted.

In the crystal of (II), the charge-assisted ammonium-N3—H⋯O(carboxyl­ate) and hy­droxy-O—H⋯O(carboxyl­ate) hydrogen-bonds, that lead to the formation of a nine-membered {⋯OCO⋯HNC_2_OH} heterosynthon, observed in (I) persist, Fig. 5 ▸(*a*). However, in (II), through the agency of having two ammonium-N—H H atoms, the second H atom bridges a neighbouring carboxyl­ate-O2 atom leading to the formation of a supra­molecular tube, as highlighted in Fig. 5 ▸(*b*). As seen from Fig. 5 ▸(*b*), the benzene rings are aligned to be proximate and, indeed, they inter­act *via* π–π stacking with the inter-centroid separation being 3.4944 (9) Å (symmetry operation: 1 − *x*, *y*, 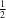 − *z*). The carboxyl­ate-O2 atom forms two hydrogen-bonds. The connections between the tubes are of the type methyl­ene- and methyl-C—H⋯O(nitro), involving both nitro-O atoms, as well as π–π stacking between benzene rings [inter-centroid separation = 3.5226 (10) Å for symmetry operation: 1 − *x*, 1 − *y*, −*z*]. A view of the unit-cell contents is shown in Fig. 5 ▸(*c*), highlighting the intra- and inter-tube π–π stacking along the *c*-axis direction.

**Figure 6:**
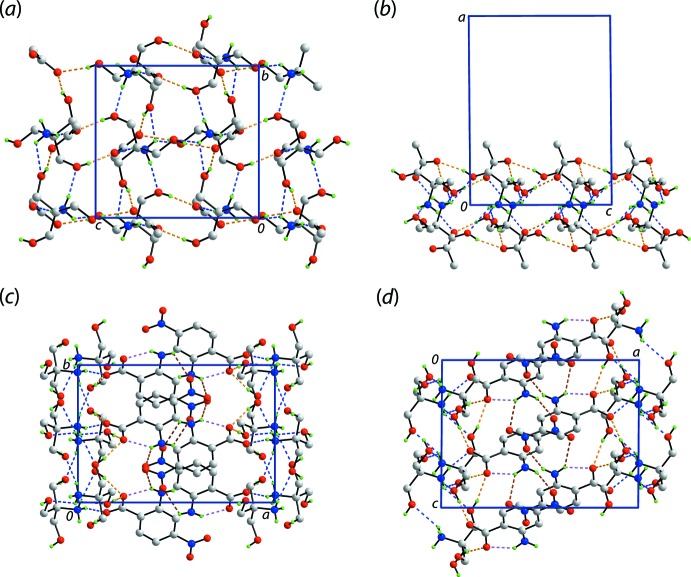
The mol­ecular packing in (III): (*a*) plan and (*b*) views of the double-layer sustained by charge-assisted ammonium-N3—H⋯O(carboxyl­ate), ammonium-N3—H⋯O(hy­droxy) (blue dashed lines) and hy­droxy-O—H⋯O(carboxyl­ate) (orange dashed lines) hydrogen bonds, and views of the unit-cell contents in projection down the (*c*) *c* axis and (*d*) *b* axis. In (*c*) and (*d*), the intra­molecular amine-N—H⋯O(carboxyl­ate) and amine-N—H⋯O(nitro) inter­actions are represented by pink and brown dashed lines, respectively. In each of (*a*)–(*d*), non-participating H atoms are omitted.

In the crystal of (III), supra­molecular double-layers in the *bc*-plane are formed as a result of charge-assisted ammonium-N3—H⋯O(carboxyl­ate), ammonium-N3—H⋯O(hy­droxy) and hy­droxy-O—H⋯O(carboxyl­ate) hydrogen-bonds. The ammonium-N3—H3*N* atom is bifurcated, forming two weak ammonium-N3—H⋯O(hy­droxy) hydrogen-bonds. A view normal to the plane of the double-layer and a side-on view are shown in Fig. 6 ▸(*a*) and (*b*), respectively. From the latter, the intra-layer region comprises the ammonium groups, each of which forms four N—H⋯O hydrogen-bonds to carboxyl­ate and hy­droxy groups on either side. Each hy­droxy group of the cation forms a hy­droxy-O—H⋯O(carboxyl­ate) hydrogen-bond with a carboxyl­ate-O atom derived from a different anion, and each accepts an ammonium-N—H atom derived from a different cation. Each carboxyl­ate-O atom forms two hydrogen-bonds, the O1 accepts hydrogen-bonds from different hy­droxy groups, and the O2 atom accept hydrogen-bonds from hy­droxy and ammonium groups. Projecting to either side of the double-layer are the nitro­benzene groups, Fig. 6 ▸(*c*) and (*d*). These provide the links to construct the three-dimensional architecture, *i.e. via* amine-N—H⋯O(nitro) inter­actions, involving both nitro-O atoms.

The obvious trend from the present study is the increase in dimensionality of the supra­molecular aggregation pattern, *i.e*. chain in (I), tube in (II) and double-layer in (III), as the number of acidic ammonium-N—H atoms increases.

## Database survey   

As indicated in the *Chemical context*, a number of ammonium salts of the anion derived from 2-amino-4-nitro­benzoic acid have now been described. The key conformational indicators for the anion are the dihedral angles formed between CO_2_/C_6_/NO_2_. The smallest dihedral angles between the CO_2_/C_6_, C_6_/NO_2_ and CO_2_/NO_2_ pairs of least-squares planes of 3.44 (14), 0.69 (11) and 3.2 (2)° are found for the anion in the salt with H_3_N^(+)^CH_2_CH_2_N^(+)^H_3_ (Smith *et al.*, 2002 ▸). Conversely, the greatest CO_2_/C_6_, C_6_/NO_2_ and CO_2_/NO_2_ dihedral angles of 26.4 (3), 12.6 (3) and 26.73 (14)°, respectively, are found in the N^(+)^H_4_ (Smith, 2014*b* ▸), *n*-Bu_2_N^(+)^H_2_ (Wardell *et al.*, 2016 ▸) and H_2_NN^(+)^H_3_ (Wardell *et al.*, 2017 ▸) salts, respectively. The respective dihedral angles in (I)–(III), described herein, fall within these ranges.

## Synthesis and crystallization   

Preparation of dimeth­yl(2-hy­droxy­eth­yl)ammonium 2-amino-4-nitro­benzoate (I). To a solution of 2-amino-4-nitro­benzoic acid (1 mmol) in methanol (10 ml) was added a solution of dimeth­yl(2-hy­droxy­eth­yl)amine (1 mmol) in methanol (10 ml). The reaction mixture was refluxed for 15 mins, and then maintained at room temperature. Crystals of (I) were collected after three days. M.p. 444–447 K. Anal. calcd.: C, 48.89; H, 5.97, N, 15.54. Found: C, 48.81; H, 5.89; N, 14.68%. IR (KBr, cm^−1^): 3500–2700 (*br*, *s*; with maxima at 3439, 3324, 3219, 2978, 2826), 1632, 1537, 1433, 1381, 1346, 1329, 1279, 1263, 1209, 1140, 1099, 1072, 1022, 918, 858, 823, 785, 731, 692, 684, 577, 513, 486.

Preparation of *tert*-but­yl(2-hy­droxy­eth­yl)ammonium 2-amino-4-nitro­benzoate (II). To a solution of 2-amino-4-nitro­benzoic acid (1 mmol) in methanol (10 ml) was added a solution of *tert*-but­yl(2-hy­droxy­eth­yl)amine (1 mmol) in methanol (10 ml). The reaction mixture was refluxed for 15 mins, and then maintained at room temperature. Crystals of (II) were collected after 3 days. M.p. 429–431 K. Anal. calcd.: C, 52.34; H, 6.76, N, 14.09. Found: C, 52.27; H, 6.89; N, 13.99%. IR (KBr, cm^−1^): 3550–2700 (*br*, *s*, with maxima at 3430, 3327, 3224, 2968, 2810), 1640, 1446, 1370, 1351, 1329, 1269, 1221 1137, 1085, 1034, 858, 8245, 739, 687, 587.

Preparation of tris­(hy­droxy­meth­yl)methyl­ammonium 2-amino-4-nitro­benzoate (III): To a solution of 2-amino-4-nitro­benzoic acid (1 mmol) in ethanol (10 ml) was added a solution of tris­(hy­droxy­meth­yl)methyl­amine (1 mmol) in ethanol (10 ml). The reaction mixture was refluxed for 10 mins, and then maintained at room temperature. Crystals of (III) were collected after two days. M.p. 460–463 K. Anal. calcd.: C, 51.06; H, 5.71, N, 14.89. Found: C, 50.94; H, 5.80; N, 14.79% IR (KBr, cm^−1^): 3700–2400 (*br*, *s*, with maxima at 3514, 3477, 3398, 3314, 3256, 3078, 2823 and 2538), 1647, 1431, 1350 1250, 1146, 1115, 1063, 1010, 872, 827, 736, 689, 596, 578 511, 484, 1549, 1356.

## Refinement   

Crystal data, data collection and structure refinement details are summarized in Table 5 ▸. Carbon-bound H atoms were placed in calculated positions (C—H = 0.95–0.99 Å) and were included in the refinement in the riding-model approximation, with *U*_iso_(H) set to 1.2–1.5*U*_eq_(C). The O- and N-bound H atoms were located from difference maps, but refined with O—H = 0.84±0.01 Å and *U*_iso_(H) = 1.5*U*_eq_(O), and with N—H = 0.86–0.88±0.01 Å and *U*_iso_(H) = 1.2*U*_eq_(N), respectively. In the refinement of (II), owing to poor agreement, a reflection, *i.e*. (0 2 0), was omitted from the final cycles of refinement.

## Supplementary Material

Crystal structure: contains datablock(s) I, II, III, global. DOI: 10.1107/S2056989018015578/hb7785sup1.cif

Structure factors: contains datablock(s) I. DOI: 10.1107/S2056989018015578/hb7785Isup2.hkl

Structure factors: contains datablock(s) II. DOI: 10.1107/S2056989018015578/hb7785IIsup3.hkl

Structure factors: contains datablock(s) III. DOI: 10.1107/S2056989018015578/hb7785IIIsup4.hkl

Supporting information file. DOI: 10.1107/S2056989018015578/hb7785Isup5.cml

Supporting information file. DOI: 10.1107/S2056989018015578/hb7785IIsup6.cml

Supporting information file. DOI: 10.1107/S2056989018015578/hb7785IIIsup7.cml

CCDC references: 1876928, 1876927, 1876926

Additional supporting information:  crystallographic information; 3D view; checkCIF report

## Figures and Tables

**Table 1:** Selected geometric data (Å, °) for (I)–(III)

Parameter	(I)	(II)	(III)
C7 O1	1.270 (2)	1.259 (2)	1.270 (2)
C7 O2	1.258 (2)	1.2678 (19)	1.264 (2)
CO_2_/C_6_	6.7 (3)	6.21 (13)	14.80 (17)
NO_2_/C_6_	6.6 (3)	3.28 (13)	6.58 (18)
CO_2_/NO_2_	11.5 (4)	2.94 (17)	9.7 (3)

**Table 2:** Hydrogen-bond geometry (Å, °) for (I)

*D*—H⋯*A*	*D*—H	H⋯*A*	*D*⋯*A*	*D*—H⋯*A*
N1—H1*N*⋯O1	0.89 (2)	1.97 (2)	2.6698 (19)	135 (2)
N1—H2*N*⋯O2^i^	0.88 (1)	2.24 (2)	3.010 (2)	146 (2)
N3—H3*N*⋯O1	0.90 (1)	1.82 (1)	2.6722 (18)	156 (2)
O5—H5*O*⋯O2	0.84 (2)	1.83 (2)	2.6731 (19)	179 (3)
C10—H10*B*⋯O5^ii^	0.98	2.48	3.406 (2)	157

Symmetry codes: (i) 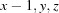; (ii) 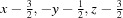.

**Table 3:** Hydrogen-bond geometry (Å, °) for (II)

*D*—H⋯*A*	*D*—H	H⋯*A*	*D*⋯*A*	*D*—H⋯*A*
N1—H1*N*⋯O1	0.87 (2)	2.00 (2)	2.665 (2)	132 (2)
N1—H2*N*⋯O5	0.89 (2)	2.17 (2)	3.058 (2)	176 (2)
N3—H3*N*⋯O1^i^	0.90 (2)	1.73 (2)	2.637 (2)	178 (2)
N3—H4*N*⋯O2^ii^	0.89 (2)	1.98 (2)	2.849 (2)	166 (2)
O5—H5*O*⋯O2^i^	0.84 (2)	1.92 (2)	2.7546 (18)	173 (2)
C11—H11*A*⋯O4^iii^	0.98	2.49	3.450 (3)	165
C12—H12*A*⋯O3	0.99	2.50	3.445 (2)	159

Symmetry codes: (i) 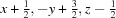; (ii) 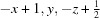; (iii) 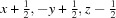.

**Table 4:** Hydrogen-bond geometry (Å, °) for (III)

*D*—H⋯*A*	*D*—H	H⋯*A*	*D*⋯*A*	*D*—H⋯*A*
N1—H1*N*⋯O1	0.88 (2)	2.02 (2)	2.678 (2)	131 (2)
N1—H1*N*⋯O3^i^	0.88 (2)	2.50 (2)	3.210 (2)	138 (1)
N1—H2*N*⋯O4^ii^	0.88 (1)	2.56 (2)	3.094 (2)	120 (2)
N3—H3*N*⋯O6^iii^	0.89 (2)	2.34 (2)	2.934 (2)	124 (1)
N3—H3*N*⋯O7^iv^	0.89 (2)	2.44 (2)	3.065 (2)	128 (2)
N3—H4*N*⋯O5^v^	0.89 (1)	2.08 (1)	2.945 (2)	165 (2)
N3—H5*N*⋯O2^vi^	0.89 (2)	1.92 (2)	2.773 (2)	160 (2)
O5—H5*O*⋯O2^vii^	0.85 (2)	1.90 (2)	2.7453 (18)	175 (2)
O6—H6*O*⋯O1^viii^	0.84 (2)	1.88 (2)	2.6993 (19)	163 (2)
O7—H7*O*⋯O1^ix^	0.84 (2)	2.07 (2)	2.8905 (18)	164 (2)

Symmetry codes: (i) 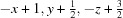; (ii) 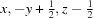; (iii) 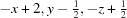; (iv) 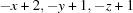; (v) 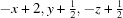; (vi) 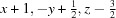; (vii) 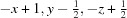; (viii) 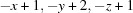; (ix) 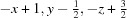.

**Table 5:** Experimental details

	(I)	(II)	(III)
Crystal data			
Chemical formula	C_4_H_12_NO^+^·C_7_H_5_N_2_O_4_^−^	C_6_H_16_NO^+^·C_7_H_5_N_2_O_4_^−^	C_4_H_12_NO_3_^+^·C_7_H_5_N_2_O_4_^−^
*M*_r_	271.27	299.33	303.27
Crystal system, space group	Monoclinic, *P*2_1_/*n*	Monoclinic, *C*2/*c*	Monoclinic, *P*2_1_/*c*
Temperature (K)	120	120	120
*a*, *b*, *c* (Å)	6.6816 (2), 22.8286 (8), 8.6570 (3)	21.1138 (5), 12.3635 (5), 13.1909 (4)	13.6269 (6), 9.4976 (3), 10.2042 (4)
β (°)	104.551 (2)	120.627 (2)	90.355 (2)
*V* (Å^3^)	1278.11 (7)	2963.02 (17)	1320.63 (9)
*Z*	4	8	4
Radiation type	Mo *K*α	Mo *K*α	Mo *K*α
μ (mm^−1^)	0.11	0.10	0.13
Crystal size (mm)	0.22 × 0.10 × 0.06	0.62 × 0.26 × 0.10	0.38 × 0.22 × 0.09

Data collection			
Diffractometer	Bruker–Nonius Roper CCD camera on κ-goniostat	Bruker–Nonius Roper CCD camera on κ-goniostat	Bruker–Nonius Roper CCD camera on κ-goniostat
Absorption correction	Multi-scan (*SADABS*; Sheldrick, 2007 ▸)	Multi-scan (*SADABS*; Sheldrick, 2007 ▸)	Multi-scan (*SADABS*; Sheldrick, 2007 ▸)
*T*_min_, *T*_max_	0.847, 1.000	0.652, 0.746	0.656, 0.746
No. of measured, independent and observed [*I* > 2σ(*I*)] reflections	9689, 2911, 2380	18353, 3406, 2349	16747, 3027, 2183
*R*_int_	0.043	0.065	0.069
(sin θ/λ)_max_ (Å^−1^)	0.649	0.651	0.651

Refinement			
*R*[*F* ^2^ > 2σ(*F* ^2^)], *wR*(*F* ^2^), *S*	0.054, 0.123, 1.07	0.050, 0.135, 1.02	0.048, 0.122, 1.04
No. of reflections	2911	3406	3027
No. of parameters	186	208	214
No. of restraints	4	5	8
H-atom treatment	H atoms treated by a mixture of independent and constrained refinement	H atoms treated by a mixture of independent and constrained refinement	H atoms treated by a mixture of independent and constrained refinement
Δρ_max_, Δρ_min_ (e Å^−3^)	0.27, −0.32	0.24, −0.33	0.31, −0.26

Computer programs: *DENZO* (Otwinowski & Minor, 1997 ▸), *COLLECT* (Hooft, 1998 ▸), *SHELXS97* (Sheldrick, 2008 ▸), *SHELXL2014* (Sheldrick, 2015 ▸), *ORTEP-3 for Windows* (Farrugia, 2012 ▸), *DIAMOND* (Brandenburg, 2006 ▸) and *publCIF* (Westrip, 2010 ▸).

## supplementary crystallographic information

### 2-Amino-4-nitrobenzoate (2-hydroxyethyl)dimethylazanium (I) Crystal data

**Table:**

C_4_H_12_NO^+^·C_7_H_5_N_2_O_4_^−^	*F*(000) = 576
*M**_r_* = 271.27	*D*_x_ = 1.410 Mg m^−^^3^
Monoclinic, *P*2_1_/*n*	Mo *K*α radiation, λ = 0.71073 Å
*a* = 6.6816 (2) Å	Cell parameters from 8678 reflections
*b* = 22.8286 (8) Å	θ = 2.9–27.5°
*c* = 8.6570 (3) Å	µ = 0.11 mm^−^^1^
β = 104.551 (2)°	*T* = 120 K
*V* = 1278.11 (7) Å^3^	Blade, yellow
*Z* = 4	0.22 × 0.10 × 0.06 mm

### 2-Amino-4-nitrobenzoate (2-hydroxyethyl)dimethylazanium (I) Data collection

**Table:**

Bruker–Nonius Roper CCD camera on κ-goniostat diffractometer	2911 independent reflections
Radiation source: Bruker–Nonius FR591 rotating anode	2380 reflections with *I* > 2σ(*I*)
Graphite monochromator	*R*_int_ = 0.043
Detector resolution: 9.091 pixels mm^-1^	θ_max_ = 27.5°, θ_min_ = 3.0°
φ & ω scans	*h* = −8→8
Absorption correction: multi-scan (SADABS; Sheldrick, 2007)	*k* = −29→29
*T*_min_ = 0.847, *T*_max_ = 1.000	*l* = −11→10
9689 measured reflections	

### 2-Amino-4-nitrobenzoate (2-hydroxyethyl)dimethylazanium (I) Refinement

**Table:**

Refinement on *F*^2^	Primary atom site location: structure-invariant direct methods
Least-squares matrix: full	Hydrogen site location: mixed
*R*[*F*^2^ > 2σ(*F*^2^)] = 0.054	H atoms treated by a mixture of independent and constrained refinement
*wR*(*F*^2^) = 0.123	*w* = 1/[σ^2^(*F*_o_^2^) + (0.0305*P*)^2^ + 1.1579*P*] where *P* = (*F*_o_^2^ + 2*F*_c_^2^)/3
*S* = 1.07	(Δ/σ)_max_ < 0.001
2911 reflections	Δρ_max_ = 0.27 e Å^−^^3^
186 parameters	Δρ_min_ = −0.31 e Å^−^^3^
4 restraints	

### 2-Amino-4-nitrobenzoate (2-hydroxyethyl)dimethylazanium (I) Special details

**Table:**

Geometry. All esds (except the esd in the dihedral angle between two l.s. planes) are estimated using the full covariance matrix. The cell esds are taken into account individually in the estimation of esds in distances, angles and torsion angles; correlations between esds in cell parameters are only used when they are defined by crystal symmetry. An approximate (isotropic) treatment of cell esds is used for estimating esds involving l.s. planes.

### 2-Amino-4-nitrobenzoate (2-hydroxyethyl)dimethylazanium (I) Fractional atomic coordinates and isotropic or equivalent isotropic displacement parameters (Å^2^)

**Table:**

	*x*	*y*	*z*	*U*_iso_*/*U*_eq_	
O1	0.16272 (19)	0.18360 (5)	0.56894 (15)	0.0234 (3)	
O2	0.3854 (2)	0.15398 (6)	0.43175 (16)	0.0276 (3)	
O3	−0.4660 (3)	−0.04973 (8)	0.2249 (3)	0.0617 (6)	
O4	−0.2616 (3)	−0.06559 (8)	0.0708 (2)	0.0553 (5)	
N1	−0.1707 (2)	0.11839 (7)	0.5634 (2)	0.0270 (4)	
H1N	−0.101 (3)	0.1513 (6)	0.593 (3)	0.032*	
H2N	−0.3042 (16)	0.1187 (10)	0.558 (3)	0.032*	
N2	−0.3090 (3)	−0.03927 (8)	0.1803 (2)	0.0385 (4)	
C1	0.0836 (3)	0.09665 (7)	0.4120 (2)	0.0188 (3)	
C2	−0.1058 (3)	0.08640 (7)	0.4521 (2)	0.0205 (4)	
C3	−0.2321 (3)	0.04031 (8)	0.3743 (2)	0.0258 (4)	
H3	−0.3606	0.0324	0.3984	0.031*	
C4	−0.1684 (3)	0.00696 (8)	0.2634 (2)	0.0275 (4)	
C5	0.0187 (3)	0.01502 (8)	0.2244 (2)	0.0283 (4)	
H5	0.0602	−0.0094	0.1491	0.034*	
C6	0.1419 (3)	0.06031 (8)	0.3006 (2)	0.0237 (4)	
H6	0.2711	0.0670	0.2765	0.028*	
C7	0.2213 (3)	0.14808 (7)	0.4763 (2)	0.0190 (3)	
O5	0.59794 (19)	0.25309 (6)	0.51573 (16)	0.0249 (3)	
H5O	0.529 (3)	0.2221 (7)	0.489 (3)	0.037*	
N3	0.1711 (2)	0.29912 (6)	0.52114 (17)	0.0189 (3)	
H3N	0.206 (3)	0.2616 (5)	0.548 (2)	0.023*	
C8	0.3578 (3)	0.33333 (8)	0.5092 (2)	0.0250 (4)	
H8A	0.4401	0.3433	0.6180	0.030*	
H8B	0.3126	0.3705	0.4520	0.030*	
C9	0.4932 (3)	0.30024 (8)	0.4229 (2)	0.0256 (4)	
H9A	0.4067	0.2848	0.3209	0.031*	
H9B	0.5960	0.3274	0.3976	0.031*	
C10	0.0140 (3)	0.29638 (9)	0.3653 (2)	0.0252 (4)	
H10A	−0.1042	0.2729	0.3768	0.038*	
H10B	0.0753	0.2783	0.2853	0.038*	
H10C	−0.0327	0.3361	0.3311	0.038*	
C11	0.0766 (3)	0.32355 (8)	0.6464 (2)	0.0251 (4)	
H11A	0.0397	0.3647	0.6223	0.038*	
H11B	0.1760	0.3208	0.7508	0.038*	
H11C	−0.0481	0.3012	0.6482	0.038*	

### 2-Amino-4-nitrobenzoate (2-hydroxyethyl)dimethylazanium (I) Atomic displacement parameters (Å^2^)

**Table:**

	*U*^11^	*U*^22^	*U*^33^	*U*^12^	*U*^13^	*U*^23^
O1	0.0236 (6)	0.0214 (6)	0.0258 (7)	−0.0023 (5)	0.0074 (5)	−0.0044 (5)
O2	0.0251 (7)	0.0280 (7)	0.0326 (7)	−0.0051 (5)	0.0126 (6)	−0.0029 (6)
O3	0.0404 (10)	0.0457 (10)	0.0971 (16)	−0.0189 (8)	0.0135 (10)	−0.0275 (10)
O4	0.0809 (13)	0.0412 (9)	0.0422 (10)	−0.0246 (9)	0.0125 (9)	−0.0190 (8)
N1	0.0216 (8)	0.0259 (8)	0.0358 (9)	−0.0034 (6)	0.0115 (7)	−0.0050 (7)
N2	0.0422 (11)	0.0233 (8)	0.0432 (11)	−0.0063 (8)	−0.0018 (8)	−0.0034 (8)
C1	0.0206 (8)	0.0158 (8)	0.0182 (8)	0.0004 (6)	0.0015 (6)	0.0023 (6)
C2	0.0206 (8)	0.0171 (8)	0.0214 (8)	0.0016 (6)	0.0009 (6)	0.0034 (7)
C3	0.0214 (9)	0.0200 (8)	0.0328 (10)	−0.0014 (7)	0.0005 (7)	0.0039 (8)
C4	0.0332 (10)	0.0169 (8)	0.0266 (9)	−0.0031 (7)	−0.0028 (8)	−0.0002 (7)
C5	0.0388 (11)	0.0197 (9)	0.0253 (9)	0.0016 (8)	0.0061 (8)	−0.0015 (7)
C6	0.0269 (9)	0.0219 (9)	0.0217 (9)	0.0030 (7)	0.0052 (7)	0.0019 (7)
C7	0.0196 (8)	0.0177 (8)	0.0185 (8)	0.0005 (6)	0.0025 (6)	0.0046 (6)
O5	0.0202 (6)	0.0237 (6)	0.0296 (7)	−0.0017 (5)	0.0041 (5)	0.0036 (5)
N3	0.0183 (7)	0.0195 (7)	0.0186 (7)	−0.0002 (6)	0.0042 (5)	−0.0013 (6)
C8	0.0218 (9)	0.0229 (9)	0.0304 (10)	−0.0054 (7)	0.0067 (7)	−0.0026 (7)
C9	0.0225 (9)	0.0256 (9)	0.0307 (10)	−0.0007 (7)	0.0106 (7)	0.0070 (8)
C10	0.0208 (8)	0.0318 (10)	0.0209 (9)	−0.0013 (7)	0.0014 (7)	−0.0028 (7)
C11	0.0288 (9)	0.0273 (9)	0.0211 (9)	0.0027 (8)	0.0099 (7)	−0.0034 (7)

### 2-Amino-4-nitrobenzoate (2-hydroxyethyl)dimethylazanium (I) Geometric parameters (Å, º)

**Table:**

O1—C7	1.270 (2)	O5—C9	1.418 (2)
O2—C7	1.258 (2)	O5—H5O	0.843 (10)
O3—N2	1.229 (3)	N3—C10	1.488 (2)
O4—N2	1.229 (3)	N3—C11	1.493 (2)
N1—C2	1.363 (2)	N3—C8	1.498 (2)
N1—H1N	0.886 (10)	N3—H3N	0.902 (9)
N1—H2N	0.881 (9)	C8—C9	1.512 (3)
N2—C4	1.474 (2)	C8—H8A	0.9900
C1—C6	1.399 (2)	C8—H8B	0.9900
C1—C2	1.413 (2)	C9—H9A	0.9900
C1—C7	1.509 (2)	C9—H9B	0.9900
C2—C3	1.410 (2)	C10—H10A	0.9800
C3—C4	1.374 (3)	C10—H10B	0.9800
C3—H3	0.9500	C10—H10C	0.9800
C4—C5	1.387 (3)	C11—H11A	0.9800
C5—C6	1.382 (3)	C11—H11B	0.9800
C5—H5	0.9500	C11—H11C	0.9800
C6—H6	0.9500		
			
C2—N1—H1N	115.2 (14)	C10—N3—C8	111.68 (14)
C2—N1—H2N	117.5 (15)	C11—N3—C8	111.54 (14)
H1N—N1—H2N	117 (2)	C10—N3—H3N	105.8 (13)
O3—N2—O4	123.56 (18)	C11—N3—H3N	107.4 (13)
O3—N2—C4	118.39 (19)	C8—N3—H3N	110.1 (13)
O4—N2—C4	118.05 (19)	N3—C8—C9	112.70 (14)
C6—C1—C2	119.52 (16)	N3—C8—H8A	109.1
C6—C1—C7	117.77 (15)	C9—C8—H8A	109.1
C2—C1—C7	122.59 (15)	N3—C8—H8B	109.1
N1—C2—C3	118.61 (16)	C9—C8—H8B	109.1
N1—C2—C1	123.25 (15)	H8A—C8—H8B	107.8
C3—C2—C1	118.14 (16)	O5—C9—C8	111.72 (15)
C4—C3—C2	119.58 (17)	O5—C9—H9A	109.3
C4—C3—H3	120.2	C8—C9—H9A	109.3
C2—C3—H3	120.2	O5—C9—H9B	109.3
C3—C4—C5	123.60 (17)	C8—C9—H9B	109.3
C3—C4—N2	117.77 (18)	H9A—C9—H9B	107.9
C5—C4—N2	118.63 (18)	N3—C10—H10A	109.5
C6—C5—C4	116.63 (18)	N3—C10—H10B	109.5
C6—C5—H5	121.7	H10A—C10—H10B	109.5
C4—C5—H5	121.7	N3—C10—H10C	109.5
C5—C6—C1	122.49 (18)	H10A—C10—H10C	109.5
C5—C6—H6	118.8	H10B—C10—H10C	109.5
C1—C6—H6	118.8	N3—C11—H11A	109.5
O2—C7—O1	123.80 (16)	N3—C11—H11B	109.5
O2—C7—C1	117.90 (15)	H11A—C11—H11B	109.5
O1—C7—C1	118.28 (15)	N3—C11—H11C	109.5
C9—O5—H5O	108.9 (16)	H11A—C11—H11C	109.5
C10—N3—C11	110.07 (14)	H11B—C11—H11C	109.5
			
C6—C1—C2—N1	177.60 (16)	C3—C4—C5—C6	−1.6 (3)
C7—C1—C2—N1	−6.6 (3)	N2—C4—C5—C6	177.51 (17)
C6—C1—C2—C3	−1.6 (2)	C4—C5—C6—C1	0.1 (3)
C7—C1—C2—C3	174.24 (15)	C2—C1—C6—C5	1.5 (3)
N1—C2—C3—C4	−179.05 (17)	C7—C1—C6—C5	−174.52 (16)
C1—C2—C3—C4	0.2 (2)	C6—C1—C7—O2	−3.1 (2)
C2—C3—C4—C5	1.5 (3)	C2—C1—C7—O2	−178.95 (15)
C2—C3—C4—N2	−177.62 (16)	C6—C1—C7—O1	175.26 (15)
O3—N2—C4—C3	−7.2 (3)	C2—C1—C7—O1	−0.6 (2)
O4—N2—C4—C3	173.39 (19)	C10—N3—C8—C9	−73.87 (19)
O3—N2—C4—C5	173.7 (2)	C11—N3—C8—C9	162.49 (15)
O4—N2—C4—C5	−5.7 (3)	N3—C8—C9—O5	−71.15 (19)

### 2-Amino-4-nitrobenzoate (2-hydroxyethyl)dimethylazanium (I) Hydrogen-bond geometry (Å, º)

**Table:**

*D*—H···*A*	*D*—H	H···*A*	*D*···*A*	*D*—H···*A*
N1—H1*N*···O1	0.89 (2)	1.97 (2)	2.6698 (19)	135 (2)
N1—H2*N*···O2^i^	0.88 (1)	2.24 (2)	3.010 (2)	146 (2)
N3—H3*N*···O1	0.90 (1)	1.82 (1)	2.6722 (18)	156 (2)
O5—H5*O*···O2	0.84 (2)	1.83 (2)	2.6731 (19)	179 (3)
C10—H10*B*···O5^ii^	0.98	2.48	3.406 (2)	157

Symmetry codes: (i) *x*−1, *y*, *z*; (ii) *x*−3/2, −*y*−1/2, *z*−3/2.

### 2-Amino-4-nitrobenzoate tert-butyl(2-hydroxyethyl)azanium (II) Crystal data

**Table:**

C_6_H_16_NO^+^·C_7_H_5_N_2_O_4_^−^	*F*(000) = 1280
*M**_r_* = 299.33	*D*_x_ = 1.342 Mg m^−^^3^
Monoclinic, *C*2/*c*	Mo *K*α radiation, λ = 0.71073 Å
*a* = 21.1138 (5) Å	Cell parameters from 8229 reflections
*b* = 12.3635 (5) Å	θ = 2.9–27.5°
*c* = 13.1909 (4) Å	µ = 0.10 mm^−^^1^
β = 120.627 (2)°	*T* = 120 K
*V* = 2963.02 (17) Å^3^	Slab, orange
*Z* = 8	0.62 × 0.26 × 0.10 mm

### 2-Amino-4-nitrobenzoate tert-butyl(2-hydroxyethyl)azanium (II) Data collection

**Table:**

Bruker–Nonius Roper CCD camera on κ-goniostat diffractometer	3406 independent reflections
Radiation source: Bruker–Nonius FR591 rotating anode	2349 reflections with *I* > 2σ(*I*)
Graphite monochromator	*R*_int_ = 0.065
Detector resolution: 9.091 pixels mm^-1^	θ_max_ = 27.6°, θ_min_ = 3.0°
φ & ω scans	*h* = −27→27
Absorption correction: multi-scan (SADABS; Sheldrick, 2007)	*k* = −16→15
*T*_min_ = 0.652, *T*_max_ = 0.746	*l* = −17→17
18353 measured reflections	

### 2-Amino-4-nitrobenzoate tert-butyl(2-hydroxyethyl)azanium (II) Refinement

**Table:**

Refinement on *F*^2^	Primary atom site location: structure-invariant direct methods
Least-squares matrix: full	Hydrogen site location: mixed
*R*[*F*^2^ > 2σ(*F*^2^)] = 0.050	H atoms treated by a mixture of independent and constrained refinement
*wR*(*F*^2^) = 0.135	*w* = 1/[σ^2^(*F*_o_^2^) + (0.0712*P*)^2^ + 0.7685*P*] where *P* = (*F*_o_^2^ + 2*F*_c_^2^)/3
*S* = 1.02	(Δ/σ)_max_ < 0.001
3406 reflections	Δρ_max_ = 0.24 e Å^−^^3^
208 parameters	Δρ_min_ = −0.33 e Å^−^^3^
5 restraints	

### 2-Amino-4-nitrobenzoate tert-butyl(2-hydroxyethyl)azanium (II) Special details

**Table:**

Geometry. All esds (except the esd in the dihedral angle between two l.s. planes) are estimated using the full covariance matrix. The cell esds are taken into account individually in the estimation of esds in distances, angles and torsion angles; correlations between esds in cell parameters are only used when they are defined by crystal symmetry. An approximate (isotropic) treatment of cell esds is used for estimating esds involving l.s. planes.

### 2-Amino-4-nitrobenzoate tert-butyl(2-hydroxyethyl)azanium (II) Fractional atomic coordinates and isotropic or equivalent isotropic displacement parameters (Å^2^)

**Table:**

	*x*	*y*	*z*	*U*_iso_*/*U*_eq_	
O1	0.38287 (6)	0.76025 (10)	0.01169 (10)	0.0301 (3)	
O2	0.29310 (6)	0.63783 (10)	−0.04670 (10)	0.0263 (3)	
O3	0.63480 (6)	0.33794 (10)	0.24135 (11)	0.0305 (3)	
O4	0.54488 (7)	0.22405 (10)	0.17418 (13)	0.0400 (4)	
N1	0.52324 (7)	0.69991 (12)	0.14202 (13)	0.0241 (3)	
N2	0.56872 (7)	0.31665 (12)	0.18823 (12)	0.0241 (3)	
C1	0.41726 (8)	0.57689 (13)	0.05554 (13)	0.0188 (4)	
C2	0.49416 (8)	0.59915 (13)	0.12014 (12)	0.0187 (4)	
C3	0.54245 (8)	0.51022 (13)	0.16275 (13)	0.0192 (4)	
H3	0.5941	0.5221	0.2073	0.023*	
C4	0.51565 (8)	0.40665 (13)	0.14053 (13)	0.0195 (4)	
C5	0.44091 (8)	0.38249 (14)	0.07707 (13)	0.0215 (4)	
H5	0.4235	0.3101	0.0624	0.026*	
C6	0.39357 (8)	0.46956 (14)	0.03667 (13)	0.0214 (4)	
H6	0.3421	0.4558	−0.0063	0.026*	
C7	0.36039 (8)	0.66455 (14)	0.00382 (13)	0.0216 (4)	
O5	0.69062 (6)	0.71809 (10)	0.29593 (11)	0.0303 (3)	
N3	0.79731 (7)	0.56819 (12)	0.45647 (12)	0.0231 (3)	
C8	0.84793 (9)	0.47891 (15)	0.53503 (16)	0.0292 (4)	
C9	0.80464 (11)	0.37480 (16)	0.5120 (2)	0.0444 (5)	
H9A	0.7623	0.3876	0.5219	0.067*	
H9B	0.8363	0.3192	0.5678	0.067*	
H9C	0.7873	0.3503	0.4313	0.067*	
C10	0.87684 (12)	0.51860 (17)	0.66040 (17)	0.0448 (5)	
H10A	0.8355	0.5298	0.6732	0.067*	
H10B	0.9032	0.5870	0.6724	0.067*	
H10C	0.9104	0.4646	0.7163	0.067*	
C11	0.91074 (10)	0.46495 (17)	0.51089 (19)	0.0402 (5)	
H11A	0.9478	0.4160	0.5696	0.060*	
H11B	0.9333	0.5355	0.5155	0.060*	
H11C	0.8916	0.4344	0.4320	0.060*	
C12	0.75766 (9)	0.54960 (16)	0.32642 (15)	0.0310 (4)	
H12A	0.7141	0.5033	0.3028	0.037*	
H12B	0.7905	0.5118	0.3049	0.037*	
C13	0.73361 (9)	0.65690 (16)	0.26214 (15)	0.0301 (4)	
H13A	0.7777	0.6992	0.2789	0.036*	
H13B	0.7046	0.6434	0.1762	0.036*	
H1N	0.4925 (9)	0.7539 (12)	0.1137 (17)	0.036*	
H2N	0.5715 (5)	0.7082 (16)	0.1885 (15)	0.036*	
H5O	0.7197 (10)	0.7625 (14)	0.3464 (15)	0.045*	
H3N	0.8263 (9)	0.6272 (11)	0.4739 (16)	0.036*	
H4N	0.7637 (8)	0.5810 (16)	0.4767 (16)	0.036*	

### 2-Amino-4-nitrobenzoate tert-butyl(2-hydroxyethyl)azanium (II) Atomic displacement parameters (Å^2^)

**Table:**

	*U*^11^	*U*^22^	*U*^33^	*U*^12^	*U*^13^	*U*^23^
O1	0.0220 (6)	0.0240 (7)	0.0377 (7)	0.0031 (5)	0.0104 (6)	0.0033 (5)
O2	0.0159 (6)	0.0339 (7)	0.0272 (6)	0.0023 (5)	0.0096 (5)	0.0055 (5)
O3	0.0197 (6)	0.0276 (7)	0.0389 (7)	0.0024 (5)	0.0112 (5)	−0.0006 (6)
O4	0.0337 (7)	0.0186 (7)	0.0580 (9)	−0.0016 (6)	0.0163 (7)	0.0032 (6)
N1	0.0169 (7)	0.0189 (8)	0.0318 (8)	−0.0002 (6)	0.0089 (6)	−0.0009 (6)
N2	0.0223 (7)	0.0237 (8)	0.0254 (7)	0.0007 (6)	0.0114 (6)	0.0000 (6)
C1	0.0181 (8)	0.0232 (9)	0.0162 (7)	0.0006 (7)	0.0096 (6)	0.0000 (6)
C2	0.0192 (8)	0.0221 (9)	0.0158 (7)	−0.0003 (7)	0.0097 (6)	−0.0004 (6)
C3	0.0160 (7)	0.0246 (9)	0.0177 (7)	−0.0009 (6)	0.0090 (6)	−0.0006 (6)
C4	0.0206 (8)	0.0194 (9)	0.0194 (7)	0.0028 (7)	0.0110 (6)	0.0018 (6)
C5	0.0222 (8)	0.0208 (9)	0.0229 (8)	−0.0042 (7)	0.0125 (7)	−0.0024 (7)
C6	0.0172 (7)	0.0294 (10)	0.0182 (7)	−0.0029 (7)	0.0095 (6)	−0.0022 (7)
C7	0.0200 (8)	0.0271 (10)	0.0174 (7)	0.0036 (7)	0.0093 (6)	0.0022 (7)
O5	0.0202 (6)	0.0296 (8)	0.0306 (7)	−0.0012 (5)	0.0053 (5)	−0.0053 (5)
N3	0.0181 (7)	0.0210 (8)	0.0295 (7)	−0.0011 (6)	0.0114 (6)	−0.0027 (6)
C8	0.0236 (8)	0.0234 (10)	0.0378 (10)	0.0060 (7)	0.0136 (8)	0.0037 (8)
C9	0.0352 (10)	0.0261 (11)	0.0740 (15)	0.0047 (9)	0.0295 (11)	0.0084 (10)
C10	0.0526 (12)	0.0396 (12)	0.0357 (11)	0.0156 (10)	0.0178 (10)	0.0129 (9)
C11	0.0247 (9)	0.0367 (12)	0.0578 (13)	0.0075 (8)	0.0200 (9)	0.0040 (10)
C12	0.0227 (8)	0.0326 (11)	0.0304 (9)	−0.0014 (7)	0.0083 (7)	−0.0104 (8)
C13	0.0250 (9)	0.0379 (11)	0.0232 (8)	−0.0032 (8)	0.0093 (7)	−0.0060 (8)

### 2-Amino-4-nitrobenzoate tert-butyl(2-hydroxyethyl)azanium (II) Geometric parameters (Å, º)

**Table:**

O1—C7	1.259 (2)	N3—C8	1.519 (2)
O2—C7	1.2678 (19)	N3—H3N	0.903 (9)
O3—N2	1.2292 (17)	N3—H4N	0.890 (9)
O4—N2	1.2262 (18)	C8—C9	1.517 (3)
N1—C2	1.353 (2)	C8—C11	1.523 (3)
N1—H1N	0.872 (9)	C8—C10	1.523 (3)
N1—H2N	0.887 (9)	C9—H9A	0.9800
N2—C4	1.473 (2)	C9—H9B	0.9800
C1—C6	1.395 (2)	C9—H9C	0.9800
C1—C2	1.424 (2)	C10—H10A	0.9800
C1—C7	1.499 (2)	C10—H10B	0.9800
C2—C3	1.407 (2)	C10—H10C	0.9800
C3—C4	1.370 (2)	C11—H11A	0.9800
C3—H3	0.9500	C11—H11B	0.9800
C4—C5	1.391 (2)	C11—H11C	0.9800
C5—C6	1.378 (2)	C12—C13	1.516 (3)
C5—H5	0.9500	C12—H12A	0.9900
C6—H6	0.9500	C12—H12B	0.9900
O5—C13	1.417 (2)	C13—H13A	0.9900
O5—H5O	0.841 (10)	C13—H13B	0.9900
N3—C12	1.494 (2)		
			
C2—N1—H1N	117.1 (13)	C9—C8—C11	111.21 (16)
C2—N1—H2N	119.3 (13)	N3—C8—C11	108.94 (15)
H1N—N1—H2N	123.4 (19)	C9—C8—C10	111.00 (17)
O4—N2—O3	123.05 (14)	N3—C8—C10	105.08 (14)
O4—N2—C4	118.44 (13)	C11—C8—C10	110.70 (16)
O3—N2—C4	118.50 (14)	C8—C9—H9A	109.5
C6—C1—C2	119.11 (14)	C8—C9—H9B	109.5
C6—C1—C7	118.37 (13)	H9A—C9—H9B	109.5
C2—C1—C7	122.50 (14)	C8—C9—H9C	109.5
N1—C2—C3	118.44 (13)	H9A—C9—H9C	109.5
N1—C2—C1	124.09 (14)	H9B—C9—H9C	109.5
C3—C2—C1	117.46 (14)	C8—C10—H10A	109.5
C4—C3—C2	120.56 (14)	C8—C10—H10B	109.5
C4—C3—H3	119.7	H10A—C10—H10B	109.5
C2—C3—H3	119.7	C8—C10—H10C	109.5
C3—C4—C5	123.22 (15)	H10A—C10—H10C	109.5
C3—C4—N2	118.23 (13)	H10B—C10—H10C	109.5
C5—C4—N2	118.54 (14)	C8—C11—H11A	109.5
C6—C5—C4	116.24 (15)	C8—C11—H11B	109.5
C6—C5—H5	121.9	H11A—C11—H11B	109.5
C4—C5—H5	121.9	C8—C11—H11C	109.5
C5—C6—C1	123.40 (14)	H11A—C11—H11C	109.5
C5—C6—H6	118.3	H11B—C11—H11C	109.5
C1—C6—H6	118.3	N3—C12—C13	109.85 (14)
O1—C7—O2	124.24 (15)	N3—C12—H12A	109.7
O1—C7—C1	117.45 (13)	C13—C12—H12A	109.7
O2—C7—C1	118.31 (15)	N3—C12—H12B	109.7
C13—O5—H5O	105.7 (15)	C13—C12—H12B	109.7
C12—N3—C8	117.35 (14)	H12A—C12—H12B	108.2
C12—N3—H3N	109.1 (12)	O5—C13—C12	112.15 (15)
C8—N3—H3N	105.3 (12)	O5—C13—H13A	109.2
C12—N3—H4N	107.8 (12)	C12—C13—H13A	109.2
C8—N3—H4N	108.4 (13)	O5—C13—H13B	109.2
H3N—N3—H4N	108.6 (18)	C12—C13—H13B	109.2
C9—C8—N3	109.71 (14)	H13A—C13—H13B	107.9
			
C6—C1—C2—N1	178.92 (14)	N2—C4—C5—C6	178.59 (13)
C7—C1—C2—N1	0.6 (2)	C4—C5—C6—C1	0.7 (2)
C6—C1—C2—C3	−0.6 (2)	C2—C1—C6—C5	−0.3 (2)
C7—C1—C2—C3	−178.87 (13)	C7—C1—C6—C5	178.09 (14)
N1—C2—C3—C4	−178.52 (14)	C6—C1—C7—O1	−173.13 (14)
C1—C2—C3—C4	1.0 (2)	C2—C1—C7—O1	5.2 (2)
C2—C3—C4—C5	−0.6 (2)	C6—C1—C7—O2	5.6 (2)
C2—C3—C4—N2	−179.45 (13)	C2—C1—C7—O2	−176.12 (14)
O4—N2—C4—C3	176.00 (14)	C12—N3—C8—C9	−58.0 (2)
O3—N2—C4—C3	−3.2 (2)	C12—N3—C8—C11	63.99 (19)
O4—N2—C4—C5	−2.9 (2)	C12—N3—C8—C10	−177.36 (15)
O3—N2—C4—C5	177.91 (14)	C8—N3—C12—C13	−159.11 (14)
C3—C4—C5—C6	−0.2 (2)	N3—C12—C13—O5	−55.18 (18)

### 2-Amino-4-nitrobenzoate tert-butyl(2-hydroxyethyl)azanium (II) Hydrogen-bond geometry (Å, º)

**Table:**

*D*—H···*A*	*D*—H	H···*A*	*D*···*A*	*D*—H···*A*
N1—H1*N*···O1	0.87 (2)	2.00 (2)	2.665 (2)	132 (2)
N1—H2*N*···O5	0.89 (2)	2.17 (2)	3.058 (2)	176 (2)
N3—H3*N*···O1^i^	0.90 (2)	1.73 (2)	2.637 (2)	178 (2)
N3—H4*N*···O2^ii^	0.89 (2)	1.98 (2)	2.849 (2)	166 (2)
O5—H5*O*···O2^i^	0.84 (2)	1.92 (2)	2.7546 (18)	173 (2)
C11—H11*A*···O4^iii^	0.98	2.49	3.450 (3)	165
C12—H12*A*···O3	0.99	2.50	3.445 (2)	159

Symmetry codes: (i) *x*+1/2, −*y*+3/2, *z*−1/2; (ii) −*x*+1, *y*, −*z*+1/2; (iii) *x*+1/2, −*y*+1/2, *z*−1/2.

### 2-Amino-4-nitrobenzoate 1,3-dihydroxy-2-(hydroxymethyl)propan-2-aminium (III) Crystal data

**Table:**

C_4_H_12_NO_3_^+^·C_7_H_5_N_2_O_4_^−^	*F*(000) = 640
*M**_r_* = 303.27	*D*_x_ = 1.525 Mg m^−^^3^
Monoclinic, *P*2_1_/*c*	Mo *K*α radiation, λ = 0.71073 Å
*a* = 13.6269 (6) Å	Cell parameters from 10585 reflections
*b* = 9.4976 (3) Å	θ = 2.9–27.5°
*c* = 10.2042 (4) Å	µ = 0.13 mm^−^^1^
β = 90.355 (2)°	*T* = 120 K
*V* = 1320.63 (9) Å^3^	Slab, orange
*Z* = 4	0.38 × 0.22 × 0.09 mm

### 2-Amino-4-nitrobenzoate 1,3-dihydroxy-2-(hydroxymethyl)propan-2-aminium (III) Data collection

**Table:**

Bruker–Nonius Roper CCD camera on κ-goniostat diffractometer	3027 independent reflections
Radiation source: Bruker–Nonius FR591 rotating anode	2183 reflections with *I* > 2σ(*I*)
Graphite monochromator	*R*_int_ = 0.069
Detector resolution: 9.091 pixels mm^-1^	θ_max_ = 27.5°, θ_min_ = 2.9°
φ & ω scans	*h* = −17→17
Absorption correction: multi-scan (SADABS; Sheldrick, 2007)	*k* = −11→12
*T*_min_ = 0.656, *T*_max_ = 0.746	*l* = −11→13
16747 measured reflections	

### 2-Amino-4-nitrobenzoate 1,3-dihydroxy-2-(hydroxymethyl)propan-2-aminium (III) Refinement

**Table:**

Refinement on *F*^2^	Primary atom site location: structure-invariant direct methods
Least-squares matrix: full	Hydrogen site location: mixed
*R*[*F*^2^ > 2σ(*F*^2^)] = 0.048	H atoms treated by a mixture of independent and constrained refinement
*wR*(*F*^2^) = 0.122	*w* = 1/[σ^2^(*F*_o_^2^) + (0.055*P*)^2^ + 0.4405*P*] where *P* = (*F*_o_^2^ + 2*F*_c_^2^)/3
*S* = 1.04	(Δ/σ)_max_ < 0.001
3027 reflections	Δρ_max_ = 0.31 e Å^−^^3^
214 parameters	Δρ_min_ = −0.26 e Å^−^^3^
8 restraints	

### 2-Amino-4-nitrobenzoate 1,3-dihydroxy-2-(hydroxymethyl)propan-2-aminium (III) Special details

**Table:**

Geometry. All esds (except the esd in the dihedral angle between two l.s. planes) are estimated using the full covariance matrix. The cell esds are taken into account individually in the estimation of esds in distances, angles and torsion angles; correlations between esds in cell parameters are only used when they are defined by crystal symmetry. An approximate (isotropic) treatment of cell esds is used for estimating esds involving l.s. planes.

### 2-Amino-4-nitrobenzoate 1,3-dihydroxy-2-(hydroxymethyl)propan-2-aminium (III) Fractional atomic coordinates and isotropic or equivalent isotropic displacement parameters (Å^2^)

**Table:**

	*x*	*y*	*z*	*U*_iso_*/*U*_eq_	
O1	0.23149 (10)	1.04393 (14)	0.76677 (13)	0.0214 (3)	
O2	0.14548 (9)	0.92617 (13)	0.61598 (12)	0.0175 (3)	
O3	0.65621 (10)	0.73923 (15)	0.52281 (13)	0.0255 (3)	
O4	0.56887 (10)	0.60326 (14)	0.39818 (13)	0.0226 (3)	
N1	0.42471 (12)	1.07055 (17)	0.72756 (15)	0.0187 (4)	
H1N	0.3741 (11)	1.100 (2)	0.7735 (18)	0.022*	
H2N	0.4832 (9)	1.086 (2)	0.7608 (19)	0.022*	
N2	0.57664 (12)	0.69917 (16)	0.47821 (14)	0.0179 (4)	
C1	0.31934 (13)	0.89761 (19)	0.61872 (16)	0.0147 (4)	
C2	0.41270 (14)	0.95131 (18)	0.65438 (16)	0.0144 (4)	
C3	0.49671 (14)	0.88243 (19)	0.60661 (16)	0.0156 (4)	
H3	0.5603	0.9150	0.6306	0.019*	
C4	0.48646 (13)	0.76770 (19)	0.52504 (17)	0.0155 (4)	
C5	0.39602 (14)	0.71363 (19)	0.48678 (17)	0.0167 (4)	
H5	0.3910	0.6348	0.4298	0.020*	
C6	0.31356 (14)	0.78010 (19)	0.53561 (17)	0.0160 (4)	
H6	0.2507	0.7448	0.5120	0.019*	
C7	0.22586 (14)	0.96075 (18)	0.66953 (16)	0.0151 (4)	
O5	0.93618 (10)	0.33420 (13)	0.11526 (12)	0.0172 (3)	
H5O	0.9100 (15)	0.357 (2)	0.0431 (13)	0.026*	
O6	0.91226 (10)	0.77428 (13)	0.16735 (13)	0.0186 (3)	
H6O	0.8659 (12)	0.820 (2)	0.201 (2)	0.028*	
O7	0.85135 (10)	0.48735 (14)	0.47852 (12)	0.0212 (3)	
H7O	0.8181 (15)	0.511 (2)	0.5444 (16)	0.032*	
N3	0.99779 (11)	0.55298 (16)	0.30064 (15)	0.0137 (3)	
H3N	1.0132 (15)	0.4938 (17)	0.3649 (15)	0.016*	
H4N	1.0077 (15)	0.6377 (13)	0.3343 (18)	0.016*	
H5N	1.0380 (12)	0.542 (2)	0.2325 (14)	0.016*	
C8	0.89311 (13)	0.53736 (18)	0.25643 (16)	0.0144 (4)	
C9	0.87415 (14)	0.38355 (18)	0.21779 (17)	0.0158 (4)	
H9A	0.8839	0.3232	0.2959	0.019*	
H9B	0.8049	0.3738	0.1894	0.019*	
C10	0.87791 (14)	0.63603 (18)	0.13984 (17)	0.0163 (4)	
H10A	0.9132	0.5982	0.0630	0.020*	
H10B	0.8072	0.6399	0.1174	0.020*	
C11	0.82764 (14)	0.5772 (2)	0.37062 (17)	0.0180 (4)	
H11A	0.8386	0.6769	0.3951	0.022*	
H11B	0.7578	0.5655	0.3457	0.022*	

### 2-Amino-4-nitrobenzoate 1,3-dihydroxy-2-(hydroxymethyl)propan-2-aminium (III) Atomic displacement parameters (Å^2^)

**Table:**

	*U*^11^	*U*^22^	*U*^33^	*U*^12^	*U*^13^	*U*^23^
O1	0.0188 (7)	0.0239 (7)	0.0215 (7)	0.0012 (6)	0.0027 (5)	−0.0081 (6)
O2	0.0149 (7)	0.0211 (7)	0.0165 (6)	0.0007 (5)	0.0004 (5)	−0.0007 (5)
O3	0.0150 (7)	0.0364 (8)	0.0250 (7)	0.0032 (6)	−0.0015 (6)	−0.0061 (6)
O4	0.0234 (8)	0.0204 (7)	0.0241 (7)	0.0033 (6)	0.0029 (6)	−0.0061 (6)
N1	0.0156 (9)	0.0203 (9)	0.0202 (8)	−0.0006 (7)	−0.0004 (7)	−0.0060 (7)
N2	0.0178 (9)	0.0195 (8)	0.0165 (8)	0.0016 (7)	0.0014 (6)	0.0008 (6)
C1	0.0164 (10)	0.0151 (9)	0.0125 (8)	0.0018 (7)	0.0014 (7)	0.0027 (7)
C2	0.0161 (10)	0.0145 (9)	0.0126 (8)	−0.0001 (7)	0.0004 (7)	0.0017 (7)
C3	0.0138 (9)	0.0193 (10)	0.0137 (8)	−0.0002 (7)	−0.0009 (7)	0.0011 (7)
C4	0.0142 (9)	0.0172 (9)	0.0150 (9)	0.0036 (7)	0.0018 (7)	0.0033 (7)
C5	0.0200 (10)	0.0136 (9)	0.0167 (9)	0.0010 (7)	0.0007 (7)	−0.0008 (7)
C6	0.0149 (9)	0.0162 (9)	0.0170 (9)	−0.0016 (7)	−0.0005 (7)	−0.0005 (7)
C7	0.0197 (10)	0.0121 (9)	0.0136 (8)	0.0008 (7)	0.0017 (7)	0.0031 (7)
O5	0.0225 (8)	0.0160 (7)	0.0132 (6)	0.0040 (5)	−0.0004 (5)	−0.0014 (5)
O6	0.0208 (8)	0.0108 (6)	0.0242 (7)	0.0015 (5)	0.0000 (6)	0.0002 (5)
O7	0.0264 (8)	0.0231 (7)	0.0143 (6)	−0.0016 (6)	0.0053 (6)	−0.0010 (5)
N3	0.0149 (8)	0.0133 (8)	0.0129 (7)	0.0010 (6)	0.0009 (6)	0.0006 (6)
C8	0.0143 (9)	0.0134 (9)	0.0156 (8)	0.0009 (7)	−0.0007 (7)	−0.0005 (7)
C9	0.0186 (10)	0.0131 (9)	0.0157 (8)	0.0004 (7)	0.0015 (7)	0.0000 (7)
C10	0.0193 (10)	0.0129 (9)	0.0167 (9)	0.0004 (7)	−0.0020 (7)	−0.0006 (7)
C11	0.0162 (10)	0.0197 (10)	0.0181 (9)	0.0005 (8)	0.0027 (7)	−0.0016 (7)

### 2-Amino-4-nitrobenzoate 1,3-dihydroxy-2-(hydroxymethyl)propan-2-aminium (III) Geometric parameters (Å, º)

**Table:**

O1—C7	1.270 (2)	O5—H5O	0.844 (9)
O2—C7	1.264 (2)	O6—C10	1.421 (2)
O3—N2	1.233 (2)	O6—H6O	0.841 (10)
O4—N2	1.228 (2)	O7—C11	1.429 (2)
N1—C2	1.366 (2)	O7—H7O	0.844 (10)
N1—H1N	0.882 (9)	N3—C8	1.501 (2)
N1—H2N	0.877 (10)	N3—H3N	0.888 (9)
N2—C4	1.473 (2)	N3—H4N	0.885 (9)
C1—C6	1.404 (3)	N3—H5N	0.894 (9)
C1—C2	1.416 (3)	C8—C11	1.520 (2)
C1—C7	1.503 (3)	C8—C10	1.528 (2)
C2—C3	1.408 (3)	C8—C9	1.535 (2)
C3—C4	1.378 (3)	C9—H9A	0.9900
C3—H3	0.9500	C9—H9B	0.9900
C4—C5	1.389 (3)	C10—H10A	0.9900
C5—C6	1.384 (3)	C10—H10B	0.9900
C5—H5	0.9500	C11—H11A	0.9900
C6—H6	0.9500	C11—H11B	0.9900
O5—C9	1.428 (2)		
			
C2—N1—H1N	117.5 (14)	C8—N3—H3N	112.3 (13)
C2—N1—H2N	117.1 (15)	C8—N3—H4N	110.4 (13)
H1N—N1—H2N	117 (2)	H3N—N3—H4N	104.7 (18)
O4—N2—O3	123.12 (16)	C8—N3—H5N	109.9 (13)
O4—N2—C4	118.36 (15)	H3N—N3—H5N	111.1 (19)
O3—N2—C4	118.51 (15)	H4N—N3—H5N	108.2 (19)
C6—C1—C2	119.25 (17)	N3—C8—C11	107.84 (14)
C6—C1—C7	118.76 (16)	N3—C8—C10	107.31 (14)
C2—C1—C7	121.97 (16)	C11—C8—C10	111.52 (15)
N1—C2—C3	118.65 (17)	N3—C8—C9	109.24 (14)
N1—C2—C1	122.93 (17)	C11—C8—C9	109.60 (15)
C3—C2—C1	118.34 (16)	C10—C8—C9	111.22 (14)
C4—C3—C2	119.80 (17)	O5—C9—C8	113.65 (15)
C4—C3—H3	120.1	O5—C9—H9A	108.8
C2—C3—H3	120.1	C8—C9—H9A	108.8
C3—C4—C5	123.28 (17)	O5—C9—H9B	108.8
C3—C4—N2	117.63 (16)	C8—C9—H9B	108.8
C5—C4—N2	119.09 (16)	H9A—C9—H9B	107.7
C6—C5—C4	116.82 (17)	O6—C10—C8	111.71 (14)
C6—C5—H5	121.6	O6—C10—H10A	109.3
C4—C5—H5	121.6	C8—C10—H10A	109.3
C5—C6—C1	122.50 (17)	O6—C10—H10B	109.3
C5—C6—H6	118.7	C8—C10—H10B	109.3
C1—C6—H6	118.7	H10A—C10—H10B	107.9
O2—C7—O1	123.17 (17)	O7—C11—C8	108.12 (15)
O2—C7—C1	118.74 (16)	O7—C11—H11A	110.1
O1—C7—C1	118.06 (16)	C8—C11—H11A	110.1
C9—O5—H5O	107.9 (15)	O7—C11—H11B	110.1
C10—O6—H6O	108.1 (16)	C8—C11—H11B	110.1
C11—O7—H7O	109.4 (16)	H11A—C11—H11B	108.4
			
C6—C1—C2—N1	175.53 (16)	C2—C1—C6—C5	0.1 (3)
C7—C1—C2—N1	−5.8 (3)	C7—C1—C6—C5	−178.55 (16)
C6—C1—C2—C3	−1.2 (2)	C6—C1—C7—O2	−14.4 (2)
C7—C1—C2—C3	177.46 (15)	C2—C1—C7—O2	166.90 (16)
N1—C2—C3—C4	−175.49 (16)	C6—C1—C7—O1	163.83 (16)
C1—C2—C3—C4	1.4 (3)	C2—C1—C7—O1	−14.8 (2)
C2—C3—C4—C5	−0.5 (3)	N3—C8—C9—O5	−59.01 (18)
C2—C3—C4—N2	−179.78 (15)	C11—C8—C9—O5	−176.96 (14)
O4—N2—C4—C3	−174.88 (15)	C10—C8—C9—O5	59.3 (2)
O3—N2—C4—C3	6.3 (2)	N3—C8—C10—O6	−49.84 (19)
O4—N2—C4—C5	5.8 (2)	C11—C8—C10—O6	68.0 (2)
O3—N2—C4—C5	−173.00 (16)	C9—C8—C10—O6	−169.27 (15)
C3—C4—C5—C6	−0.5 (3)	N3—C8—C11—O7	−58.12 (18)
N2—C4—C5—C6	178.72 (15)	C10—C8—C11—O7	−175.70 (14)
C4—C5—C6—C1	0.7 (3)	C9—C8—C11—O7	60.70 (18)

### 2-Amino-4-nitrobenzoate 1,3-dihydroxy-2-(hydroxymethyl)propan-2-aminium (III) Hydrogen-bond geometry (Å, º)

**Table:**

*D*—H···*A*	*D*—H	H···*A*	*D*···*A*	*D*—H···*A*
N1—H1*N*···O1	0.88 (2)	2.02 (2)	2.678 (2)	131 (2)
N1—H1*N*···O3^i^	0.88 (2)	2.50 (2)	3.210 (2)	138 (1)
N1—H2*N*···O4^ii^	0.88 (1)	2.56 (2)	3.094 (2)	120 (2)
N3—H3*N*···O6^iii^	0.89 (2)	2.34 (2)	2.934 (2)	124 (1)
N3—H3*N*···O7^iv^	0.89 (2)	2.44 (2)	3.065 (2)	128 (2)
N3—H4*N*···O5^v^	0.89 (1)	2.08 (1)	2.945 (2)	165 (2)
N3—H5*N*···O2^vi^	0.89 (2)	1.92 (2)	2.773 (2)	160 (2)
O5—H5*O*···O2^vii^	0.85 (2)	1.90 (2)	2.7453 (18)	175 (2)
O6—H6*O*···O1^viii^	0.84 (2)	1.88 (2)	2.6993 (19)	163 (2)
O7—H7*O*···O1^ix^	0.84 (2)	2.07 (2)	2.8905 (18)	164 (2)

Symmetry codes: (i) −*x*+1, *y*+1/2, −*z*+3/2; (ii) *x*, −*y*+1/2, *z*−1/2; (iii) −*x*+2, *y*−1/2, −*z*+1/2; (iv) −*x*+2, −*y*+1, −*z*+1; (v) −*x*+2, *y*+1/2, −*z*+1/2; (vi) *x*+1, −*y*+1/2, *z*−3/2; (vii) −*x*+1, *y*−1/2, −*z*+1/2; (viii) −*x*+1, −*y*+2, −*z*+1; (ix) −*x*+1, *y*−1/2, −*z*+3/2.
